# Imaging of skin microvessels with optical coherence tomography: potential uses in port wine stains

**DOI:** 10.3892/etm.2012.711

**Published:** 2012-09-17

**Authors:** YANG ZHOU, DAIQIANG YIN, PING XUE, NAIYAN HUANG, HAIXIA QIU, YING WANG, JING ZENG, ZHIHUA DING, YING GU

**Affiliations:** 1 The Department of Laser Medicine, The PLA General Hospital, Beijing 100853;; 2 Department of Information and Electronics, Beijing Institute of Technology, Beijing 100081;; 3 Department of Physics, Tsinghua University, Beijing 100084;; 4 Optical Engineering State Key Laboratory of Modern Optical Instrumentation, Zhejiang University, Zhejiang 310058, P.R. China

**Keywords:** optical coherence tomography, photodynamic therapy, port wine stain, spectrophotometer

## Abstract

The knowledge of vascular structures of port wine stains (PWSs) may be useful to select treatment doses and improve therapeutic efficacy. Biopsies are impractical to implement, therefore, it is necessary to develop non-invasive techniques for morphological evaluation. This study aimed to evaluate the application of a novel optical coherence tomography (OCT) system to characterize the vascular structures of PWSs. First, OCT images were obtained from the skin of healthy rabbit ears and compared with the histopathological images. Second, OCT was used to document the differences between PWS lesions and contralateral normal skin; the size and depth of the vascular structures of two clinical types of PWSs were measured and statistically analyzed. The dermal blood vessels of healthy rabbit ears were clearly distinguished from other tissue. There was no statistical difference between the vascular diameter or depth measured by OCT images and histopathological sections (P>0.05). The OCT images of the PWSs could be distinguished from normal skin. There was no statistical difference in the depth of vessels between the purple-type and the proliferative-type PWSs (P>0.05), while there was statistical difference in the diameter of vessels between them (P<0.01). Therefore, OCT is a promising, real-time, *in vivo* and non-invasive tool with which to characterize the vascular structures of PWSs.

## Introduction

A port wine stain (PWS) is a congenital vascular malformation characterized pathologically by ectasia of superficial dermal capillaries and clinically by persistent macular erythema (1,2).

According to the color of PWS lesions and the degree of capillary dilation, pathologically, PWS is divided into 3 types: i) the pink type, the color of the lesions includes pink, bright red and dark red and the diameter of the dilated capillary is approximately 50–80 μm; ii) the purple type, the color of the lesions is prunosus, including light purple and dark purple and the diameter of the dilated capillary is approximately 120–150 μm; iii) the proliferative type, the color of the lesions is prunosus, the lesion surface is slightly thickened or even nodular and the diameter of the dilated capillary is greater than 150 μm. The vascular depth of all three types is approximately 100–1000 μm (3–5).

Photodynamic therapy (PDT) has become one of the most effective treatments for PWS at present (6), but there remain certain challenges. For example, various clinical outcomes are achieved within the same clinical type after the same doses of PDT, as the size and depth of the dilated capillaries vary from type to type, even in the same type of lesion or in different lesions of one patient. Knowledge concerning the vascular structure of PWSs may aid in the selection of treatment doses and improve the therapeutic effect of PDT. Biopsies are invasive and may cause side effects that make follow-up studies difficult, thus the development of non-invasive techniques for morphological assessment has important clinical significance.

Optical coherence tomography (OCT) is a high speed, high-resolution, non-invasive technique capable of generating cross-sectional images of tissue microstructure. OCT uses low coherent laser light to localize backscattering events within a specimen (similar to ultrasonography) (7). Skin is a naturally high light scattering non-transparent medium. The detection depth depends on the wavelength; the maximum penetration depth in skin is approximately 2 mm when using a l300-nm band. In the last 20 years, OCT has become a reliable method for living tissue imaging (8).

OCT has shown potential in the characterization and diagnosis of a variety of dermatological disorders, including bullous diseases, malignant melanoma, scabies, psoriasis and basal cell carcinomas (9–14). OCT also has been used in PWS. For example, Ziolkowska *et al* (15) presented one OCT image of a PWS with ectatic vessels on the cheek. Bazant-Hegemark *et al* (16) used an unsupervised, real-time classification algorithm to classify 96 synthesized test images and 7 clinical images of PWS. However, few reports have identified the vascular image features of PWS patients nor have identified the main features of other similar structures in OCT images. Thus, strong support for the clinical diagnosis and treatment of PWS using OCT is lacking.

In a previous study (17) we obtained the structural parameters of PWSs including epidermal thickness, diameter and depth of dilated blood vessels by comparing the OCT image of PWS lesions and contralateral normal skin in 41 patients, but we failed to obtain the corresponding histopathological sections.

In the present study, an OCT system was first used to detect rabbit ear skin structures and obtain certain parameters including vascular diameter, shape and location. Histopathological images were compared with the OCT images to identify clinicopathological correlations. Second, the OCT system was used on PWS patients to detect the size and depth of their vascular structures so as to provide vascular information for the objective diagnosis of PWS pathological type, selection of optimal clinical treatment dosages and prediction of therapeutic effect.

## Materials and methods

### Experimental instrument

A time domain OCT system (provided by Beijing Newraysing Laser Tech Co. Ltd., Beijing, China) with an optic Michelson interferometer with a broadband superluminescent diode (SLED) light source (center wavelength 1310 nm; maximum output power, 10 mW; bandwidth, 70 nm) was utilized. This OCT system records 4 frames per sec with a detection depth of ∼1 mm and an axial and transverse resolution of 10 and 9 μm, respectively, in skin tissue. The scanning range is 2.85×2.5 mm (axial x lateral) and image size is 400×400 pixels (axial x lateral). Signal-noise ratio is 108 dB.

### Detection using rabbit ears by OCT

Two New Zealand rabbits were used, weighing ∼0.5–1 kg. The experimental procedure was performed with the rabbits under deep anesthesia using a 3:2 ratio mixture of xylazine (20 mg/ml) and ketamine hydrochloride (100 mg/ml; 0.5ml/kg, intramuscular injection). Repeat injections were administered, as required, to keep the rabbits under deep anesthesia. Ear skin was coated with depilatory cream for 3 min, then was gently removed with saline tampon. A total of 12 test sites were documented with digital photographs. The skin was pretreated with glycerol to substantially increase the penetration depth of light through the skin and enhance differentiation between skin layers by reducing light scattering in the tissue (18–21). The hand-held OCT detector was placed directly on the skin with mild pressure and images were obtained by scanning from the side of the preparation. Each test site was scanned three times. The structural parameters, including diameter and depth of dilated blood vessels in OCT images, were determined by a measuring tool embedded in the image processing software (physical distance or depth was obtained by dividing optical depth by the bulk index of refraction of the tissue). The average of the three measurements was calculated as the mean. All OCT measurements were performed by the same investigator. The biopsy specimens were obtained within 5 min after the test, fixed in 10% buffered formalin, processed in paraffin, sectioned and stained with hematoxylin and eosin. Histopathological images were compared with the OCT images to assess the degree of their correspondence.

### Detection of PWS by OCT

The study was approved by the PLA Postgraduate Medical School ethics board. All animal experiments were carried out in accordance with the guidelines of the Animal Care and Use Committee of PLA Postgraduate Medical School. A total of 85 cases of Chinese patients with PWS on the face and neck were recruited from the outpatients of the Department of Laser Medicine, General Hospital between May 2010 and October 2011, and informed consent was obtained. Among the 81 PWS patients, 53 cases with 77 test sites belonged to the purple type and 28 cases with 44 test sites belonged to the proliferative type. Patients of the purple type were aged from 1 to 28 years, with an average age of 6.06±7.61, and patients of the proliferative type were aged from 15 to 28, average age 21.23±3.32. There was a statistical difference in age between the two types (P<0.01). The exclusion criteria were as follows: history of treatment; history of significant hematological, cardiac, hepatic, or renal disease; history of skin disease likely to interfere with the study; pregnant or breast feeding; with scar constitution; with other hemangioma or syndrome.

### Procedure and methods

Test sites were documented with digital photographs. The test sites including PWS lesions and contralateral or near normal skin (the latter was used as control) were pretreated with glycerol, then detected by OCT. The average of the three measurements was calculated corresponding to the mean. All OCT measurements were performed by the same investigator.

### Statistics

Quantitative variables were expressed as means ± SD. Data were analyzed with the statistical software SPSS 13.0. A two-tailed Student’s t-test for independent samples was used to analyze the measurement data. P<0.05 was considered to indicate a statistically significant result.

## Results

### Appearance of the vascular structure

**Figure 1:**
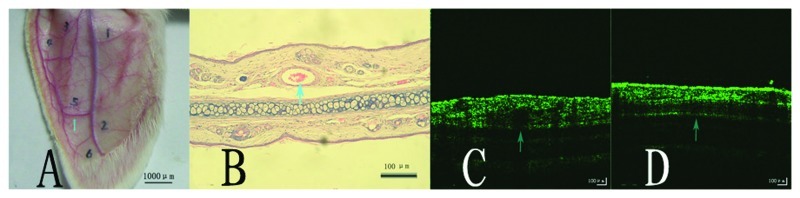
(A) Image of a rabbit ear and (B) corresponding pathological image (hematoxylin and eosin stain, magnification, x100). OCT image of (C) the transverse section of vessel and (D) the longitudinal section of vessel. Vessel is marked by an arrow.

The dermal blood vessels were distinguished clearly from normal tissue by the OCT system (Fig. 1C and D, a dark round or horizontal band area is visible, corresponding to a vascular structure), and the size and depth of the vascular structures were measured precisely. The diameter and depth of blood vessels were 238.75±79.90 and 148.92±45.73 μm, respectively, as measured by OCT images, 179.58±78.441 and 186.75±43.88 μm, respectively as measured by corresponding histopathological sections. There was no statistical difference in vascular diameter and depth (P>0.05) between the results measured by OCT images and by histopathological sections. A good correlation of vascular structures was found between the images of the histopathological sections of the rabbit ears (Fig. 1B, a blood-filled vessel lined with flattened endothelial cells) and the OCT images (Fig. 1C and D).

### Appearance of the skin structure

**Figure 2:**
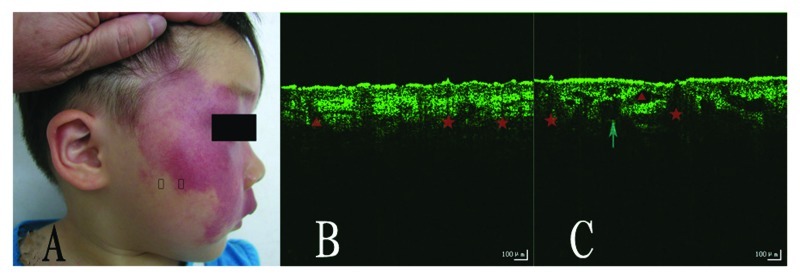
(A) Image of a patient with PWS of the purple type. OCT image of (B) normal skin and (C) the PWS lesion. The test sites are marked by rectangles, the vessel is marked by an arrow, the sebaceous glands and hair follicles are marked by a triangle and star, respectively. PWS, port wine stain; OCT, optical coherence tomography.

**Figure 3:**
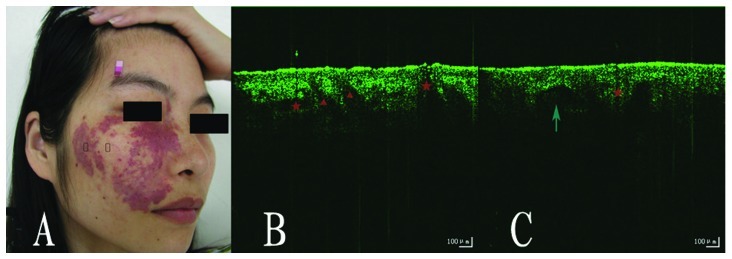
(A) Image of a patient with PWS of the proliferative type. OCT image of (B) normal skin and (C) the PWS lesion. The test sites are marked by rectangles, the vessel is marked by an arrow, the sebaceous glands and hair follicles are marked by a triangle and star, respectively. PWS, port wine stain; OCT, optical coherence tomography.

The epidermal layer, dermal layer and skin appendages, including hair follicles and sebaceous glands, were clearly visible from the OCT images (Fig. 2 and 3B and C). We also could distinguish PWS lesions from normal skin. Compared to normal skin, we observed that the stratum corneum (horny layer) was discontinuous, the dermo-epidermal border was frequently blurred, and the number of sebaceous glands and dilation of blood vessels were increased in the OCT images of PWS lesions. We obtained the structural parameters of patients with PWS. The dilated vascular depth and diameter measured by OCT images were 226.89±61.14 and 125.63±19.09 (the purple type), 195.59±59.45 and 193.93 ±32.43 (the proliferative type). There was no statistical difference in the depth of vessels between the two types (P>0.05), while there was a statistical difference in the diameter of the vessels between them (P<0.01) (Table I).

## Discussion

The average diameter of dilated capillaries in patients with PWS is usually greater then 50 μm, in contrast to a vessel diameter of 10 μm in normal skin (17–21). This OCT system has an axial resolution of l0 μm, thus we could not visualize the normal skin capillaries while it was possible to identify the dilated capillaries in the papillary layer of the PWSs. Vessels in PWS lesions may be located anywhere from approximately 150–750 μm below the skin surface, thus this OCT system with an in-depth penetration of 1 mm, was able to basically achieve the requirements of this study.

In the present study the test sites were pretreated with glycerol. OCT images distinguished between epidermal and dermal layers, and dilated capillaries and skin appendages including hair follicles and eccrine ducts were identified in the surrounding tissues.

The vascular structures commonly appeared as signal-poor round or oval areas sharply demarcated in the papillary structures of the dermis. Since the absorption coefficient (μ_a_) in the epidermis, dermis and blood is 23, 2.4 and 266 l/mol. cm, respectively (22), beneath the large diameter vessels, an ‘optical shadow’ is evident relative to the surrounding tissues due to the absorption of light by hemoglobin (23–25) .

Similar to the vascular structures, eccrine ducts are also signal-poor round or oval areas sharply demarcated in the papillary layer. The most marked difference is that the grayscale value under eccrine ducts is higher than that of the adjacent tissues at the same depth (26). This demonstrates that eccrine ducts do not scatter or absorb light. As a result, signal intensity is relatively undiminished after passing through these structures.

By comparison with the OCT images of normal skin, we observed that the stratum corneum (horny layer) succession was intermittent and the dermo-epidermal border was frequently blurred in PWS lesions; the reason for which remains unclear. The number of sebaceous glands and dilated blood vessels were increased, consequently the lesion surface of patients with PWSs is oily in clinical examination. OCT images demonstrated a clear correlation to clinical performance.

The mean vascular depth of vessels in the PWSs measured by OCT images in our study was 226.89±61.14 μm in the purple type and 195.59±59.45 μm in the proliferative type, respectively. There was no statistical difference between them. However, Barsky *et al* (5) and Zhou *et al* (27) obtained a mean vessel depth of 0.46±0.1 and 0.45±0.2 mm, respectively, through assessment of pathological sections (5). The difference may be due to the limitation of OCT penetration, since the blood vessels in deep sites cannot be measured and calculated, or due to dehydration of pathological sections. Therefore, the mean vessel depth measured by pathological sections is deeper than that by OCT images. The mean vascular diameter of the PWSs in our study was 125.63±19.09 μm in the purple type and 193.93±32.43 μm in the proliferative type, respectively. There was statistical difference between them. The mean blood vessel diameter was greater than that of other studies [the mean blood vessel diameter of a typical biopsy and confocal microscopy study was 87.72±3.21 μm (4)]. The result of our previous study (17) was 94.61±20.09 μm. The explanation for this might be collapse of blood vessels *ex vivo* and dehydration of pathological sections, so that the mean blood vessel diameter measured on pathological sections was smaller than that on OCT images *in vivo*. Moreover, in the previous studies, the clinical types of PWSs were not classified; the vessels assessed also included those of the pink type with a smaller blood vessel diameter.

In addition, we observed a number of gaps in the dermis, which appeared as regular and relatively small linear structures of poor signal quality (breadth was usually below 50 μm), and may represent lymphatic vessels or small blood vessels (15). In the papillary dermis, the outside diameters of blood vessels are generally 17±22 μm, whereas lymphatic vessels may reach up to 60 μm in diameter (28). Taking into account that these structures are present in both PWS lesions and normal skin, the authors agree that they are lymphatic vessels or small blood vessels. We also observed a number of gaps in the dermis, which appeared as irregular and relatively large linear structures of low lucidity (breadth was usually over 50 μm). In previous studies these structures have been ascribed to lymphatic vessels or blood vessels or areas of less dense collagen tissue (28–30). Considering that these structures are mainly present in PWS lesions and there is no report concerning abnormal lymphatic vessels or areas of less dense collagen tissue structures in PWS lesions, the authors presume they are blood vessels. However, if they are blood vessels, then the OCT signal below should be attenuated due to the strong absorption of hemoglobin. The absence of such a signal attenuation might be due to the fact that the diameter of the blood vessels is not large enough to absorb sufficient photons so does not produce an ‘optical shadow’.

The present study indicates that OCT clearly distinguishes dermal blood vessels in normal tissue, and distinguishes PWS lesions from normal skin. In addition, OCT accurately measures vascular diameter and depth of PWSs, which aids the objective diagnosis of PWS pathological types, the selection of optimal clinical treatment dosages and the prediction of treatment effects. Due to the superiority of this real-time, *in vivo*, discomfort-free system, OCT may be widely applied in the clinic. In subsequent research, the authors will expand the number of cases, and further observe the OCT images of PWSs following PDT treatment.

## Figures and Tables

**Table I:** Vascular depth and diameter of vessels from patients with purple- and proliferative-type PWS measured by OCT images.

Group	Depth of dilated blood vessels (mean ± SD in μm)	Diameter of dilated blood vessels (mean ± SD in μm)
Purple type	226.89±61.14	125.63±19.09
Proliferative type	195.59±59.45	193.93±32.43^a^

a P<0.01. PWS, port wine stain; OCT, optical coherence tomography.
